# Supplementary material for: Conditional chemoconnectomics (cCCTomics) as a strategy for efficient and conditional targeting of chemical transmission
Source: eLife. 2024 Apr 30;12:RP91927. doi: 10.7554/eLife.91927 (PMC11060718; doi:10.7554/eLife.91927)
Supplement: Figure 3—source data 3. [file elife-91927-fig3-data3.zip › Figure 3 source data3 part 2.zip!/Figure 3-Source data 3-part2/1025-13-CRISPResso_Report_ack040l3.zip!/CRISPResso_on_1025_15/3b.Insertion_deletion_substitutions_size_hist.pdf]

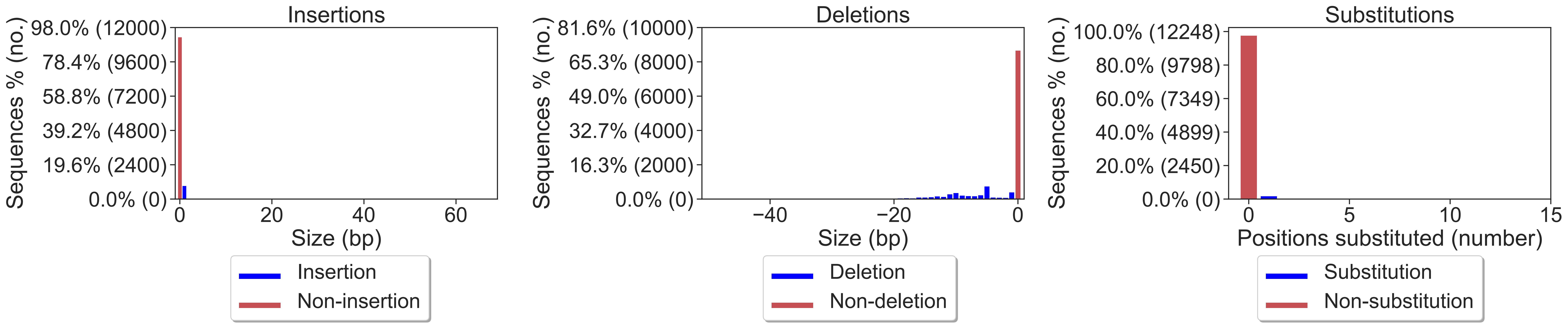
Insertions
Sequences % (no.)
98.0% (12000)
78.4% (9600)
58.8% (7200)
39.2% (4800)
19.6% (2400)
0.0% (0)
0
20
40
60
Size (bp)
Insertion
Non-insertion
Deletions
Sequences % (no.)
81.6% (10000)
65.3% (8000)
49.0% (6000)
32.7% (4000)
16.3% (2000)
0.0% (0)
−40
−20
0
Size (bp)
Deletion
Non-deletion
Substitutions
Sequences % (no.)
100.0% (12248)
80.0% (9798)
60.0% (7349)
40.0% (4899)
20.0% (2450)
0.0% (0)
0
5
10
15
Positions substituted (number)
Substitution
Non-substitution
